# From Fringe to the Mainstream: How ETD MS Brought O-GlcNAc to the *Masses*

**DOI:** 10.1016/j.mcpro.2024.100859

**Published:** 2024-10-15

**Authors:** Namrata D. Udeshi, Gerald W. Hart, Chad Slawson

**Affiliations:** 1 Broad Institute of MIT and Harvard University, Cambridge, Massachusetts, USA; 2 Department of Biochemistry and Molecular Biology, CCRC, University of Georgia, Athens, Georgia, USA; 3 Department Biochemistry, University of Kansas Medical Center, Kansas City, Kansas, USA

**Keywords:** O-GlcNAc, ETD, PTM, mass spectrometry, glycosylation

## Abstract

O-GlcNAcylation was identified in the 1980s by Torres and Hart and modifies thousands of cellular proteins, yet the regulatory role of O-GlcNAc is still poorly understood compared to the abundance of mechanistic information known for other cycling post-translational modifications like phosphorylation. Many challenges are associated with studying O-GlcNAcylation and are tied to the technical hurdles with analysis by mass spectrometry. Over the years, many research groups have developed important methods to study O-GlcNAcylation revealing its role in the cell, and this perspective aims to review the challenges and innovations around O-GlcNAc research and chronicle the work by Donald F. Hunt and his laboratory, particularly in development of ETD and its application to this field of research.

Despite the discovery of the O-GlcNAc modification 40 years ago (1) and the existence of over 4000 publications on the topic, the biology and analysis of this post-translational modification (PTM) has continued to perplex and beguile the scientific community due to a number of challenges associated with its study. O-GlcNAc is a single N-acetylglucosamine sugar attached to serine/threonine residues in cytoplasmic, nuclear, and mitochondrial proteins. The sugar is processed by a dual enzyme system: O-GlcNAc transferase (OGT), which attaches the sugar to proteins using the metabolite UDP-GlcNAc, and O-GlcNAcase (OGA), which removes the modification. One key challenge is understanding how this dual enzyme system precisely controls processing of O-GlcNAc on the right proteins at the right time (2). Furthermore, the expression of OGT and OGA are interconnected, with each enzyme’s expression oscillating in response to changes in O-GlcNAc levels (3). Another challenge arises from the fact that O-GlcNAc is present on proteins that regulate numerous biological functions, and as a result, pharmacologic or genetic manipulation of OGT or OGA can lead to pleiotropic effects on the cell. This complexity complicates the interpretation of data regarding the regulation of specific proteins and pathways (4). Furthermore, promiscuity of these enzymes for substrates makes studying O-GlcNAc site-specific regulation difficult. Moreover, both OGA and OGT are essential, and deletion of either is lethal (5, 6). Beyond mechanistic interpretation, identification of O-GlcNAcylated proteins and modification sites presents significant hurdles due to the low stoichiometry of O-GlcNAc on proteins and the highly-labile nature of O-GlcNAc modified peptides during conventional LC-MS/MS analysis. Although these complexities are still not fully understood, significant advances in O-GlcNAc biology have been driven by mass spectrometry innovations developed in Donald Hunt’s laboratory at the University of Virginia. While many research groups have contributed to the study of protein O-GlcNAcylation, this perspective aims to highlight the pivotal role of Donald F. Hunt in advancing the field of O-GlcNAc research.

The discovery of O-GlcNAc is a tale of having the right people and technologies coalescing at the right time. The story begins with the Jerry Hart laboratory attempting to characterize terminal sugars on the membranes of lymphocytes. While using a nuclear protein extract to control for beta-galactosidase labeling of membrane glycoproteins, they found extensive labeling of the nuclear extract (1). Further characterization revealed that these glycoproteins contained a single O-GlcNAc residue. This discovery was problematic because it contradicted the current glycoprotein dogma that glycoproteins only contained complex oligosaccharides and were exclusively found on membranes and secreted proteins. While glycobiologists were excited about the finding, many others chalked up the discovery as a minor, potentially inconsequential anomaly in lymphocytes. Thus, the small and nascent research groups studying O-GlcNAc faced the enormous tasks of (1) demonstrating that the modification was in multiple cell types, (2) identifying sites of modification, (3) isolating and characterizing the O-GlcNAc transferase and O-GlcNAcase (the O-GlcNAc writer and eraser enzymes, respectively), (4) proving the modification was abundant and dynamic, and (5) showing that O-GlcNAcylation could affect specific cellular functions. Although these groups were wildly successful, by the late 2000’s, O-GlcNAc research was again at an impasse. In the Hart laboratory, we had established numerous biological functions disrupted by changes in O-GlcNAc. However, the prevailing view within the broader scientific community remained that O-GlcNAc was a niche post-translational modification impacting only a few hundred proteins. Although we argued that O-GlcNAcylation was both abundant and biologically significant, several technical challenges severely hindered the mapping of O-GlcNAc sites using mass spectrometry. As a result, our research was somewhat at a standstill, particularly following our published finding that OGT was associated with the mitotic spindle and the mitotic kinase Aurora B, while over-expression of OGT induced aneuploidy in cells (7, 8). We were confident that O-GlcNAcylation of the mitotic spindle was both abundant and dynamic, but we required a method to discover O-GlcNAcylation sites from enriched spindles, rather than from purified proteins.

That’s when Jerry Hart spoke with Donald Hunt. Don had a solution.

## ETD was a Breakthrough for the Study of O-GlcNAc

At the time, collision-induced dissociation (CID) was the most commonly used MS fragmentation method for sequencing peptides and identifying sites of post-translational modification (9). As such, O-GlcNAc site detection was plagued by two major problems: 1) The sugar modification is highly labile under CID and although a diagnostic ion indicating loss of the N-acetylglucosamine is detected with CID, assigning the sugar to the correct amino acid is near impossible (10, 11) and 2) the O-GlcNAcylated peptide was hard to identify in a background of highly-abundant non-modified peptides and the signal is often suppressed by co-eluting unmodified peptides (12). Though CID had been used to identify a small number of O-GlcNAc-modified proteins, it could not be used to identify low abundant sites nor characterize the exact sites of modification because it promotes the complete loss of the sugar from serine and threonine residues upon collisional dissociation (13) hampering the identification of O-GlcNAc sites by search algorithms.

Electron transfer dissociation (ETD) was introduced by Donald Hunt’s laboratory in 2004 (14) for LC-MS/MS analysis of complex mixtures and overcame limitations of CID for the analysis of labile PTMs. ETD employs ion/ion chemistry by reacting radical anions of fluoranthene with protonated peptides in the gas phase on a millisecond timescale, making it compatible with online LC-MS/MS. ETD showed great promise for analysis of phosphorylated peptides (14, 15, 16), and therefore, would also be highly-amenable to the analysis of O-GlcNAcylated peptides.

**Fig. 1:**
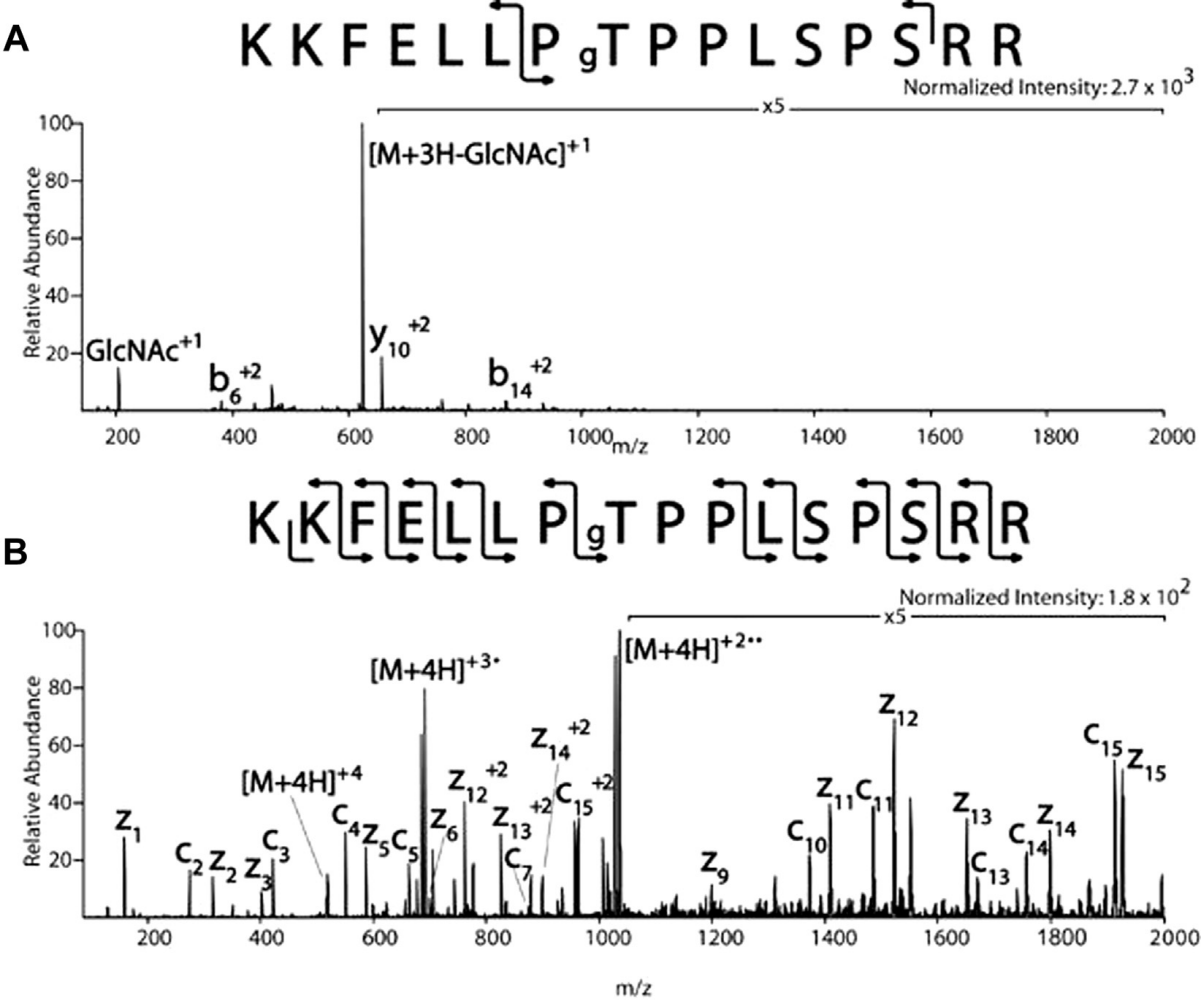
**MS/MS spectra of a synthetic O-GlcNAc containing peptide, provided by Jerry Hart to Donald Hunt ca. 2005.** This figure has been republished from Mikesh *et al.* (16) with permission. The data were collected using the original Thermo Electron LTQ ion trap mass spectrometer, which was modified to perform ETD in Hunt’s lab. *A*, a CID spectrum shows the loss of the O-GlcNAc oxoniom ion at m/z 204 and the corresponding charge reduced (M+3H) +3 product ion with a loss of 203 (GlcNAc). *B*, the ETD spectrum shows nearly complete coverage of the c and z-type ion series, with no detectable loss of GlcNAc in the ETD spectrum of the synthetic peptide.

To demonstrate the effectiveness of ETD for mapping GlcNAc sites, Jerry Hart provided the Hunt lab with a synthetic O-GlcNAc peptide to analyze by both CID and ETD (Fig. 1). The CID spectrum showed the expected diagnostic O-GlcNAc oxonium ion at m/z 204 with the corresponding charge reduced product ion that lost 203 Da (GlcNAc) (16) (Fig. 1*A*). The CID spectrum resulted in poor sequence coverage of b- and y-type ions, while the corresponding ETD spectrum of the same peptide yielded a complete c- and z-fragment ion series with no observable loss of the GlcNAc moiety (Fig. 1*B*).

## Methods for Enrichment of O-GlcNAcylation

ETD was proving to be a breakthrough technology for the identification of O-GlcNAc sites, but the detection of low-abundant O-GlcNAc modified peptides in complex mixtures still remained a major hurdle. At the time, highly efficient methods existed for the enrichment of post-translational modifications like phosphorylation (15, 17). However, a sensitive and specific method for the enrichment of O-GlcNAcylated peptides from complex mixtures was severely lacking. Beta Elimination followed by Michael Addition of DTT (BEMAD) was developed in 2002 to identify formally O-GlcNAcylated sites through removal of the sugar and replacement with a stable affinity handle that can be used for enrichment (12, 18). A limitation of BEMAD is that it removes the O-GlcNAc moiety, necessitating complementary verification. Additionally, BEMAD also results in the removal of O-phosphorylation sites and therefore requires a portion of the sample to be pre-treated with a glycosidase or dephosphorylated before analysis. Immunoaffinity purification of O-GlcNAc-modified proteins proved challenging with antibodies like CTD110.6 and RL2 (19, 20) because they suffered from low binding affinity and specificity. Wheat germ agglutinin (WGA) lectin columns had limited binding affinity for O-GlcNAc, required large amounts of sample input, and bound strongly to other glycans, which compromised specificity (21). Another common method leveraged mutant galactosyltransferase (GalT1) to label GlcNAcylated proteins with a galactose analog followed by attachment of biotin to O-GlcNAc-modified peptides. This enabled enrichment using streptavidin and subsequent analysis by ETD, but at the time the method identified few sites when applied in neurons (22).

Around 2009, recognizing ETD was emerging as a leading tool for site mapping, Jerry Hart approached Don Hunt to explore a full-fledged collaboration. Since their initial interaction at an ASBMB conference in 2003, where they had discussed innovative methods for mapping O-GlcNAc sites, Jerry now had a well-defined objective in mind. The goal was to apply ETD for mapping O-GlcNAc sites from complex mixtures to gain further mechanistic insight into the roles of the modification in cellular signaling. It was clear to us that ETD alone would not be sufficient to achieve this goal; a sensitive and specific enrichment method for O-GlcNAcylated peptides was also needed. To address this challenge, Zihao Wang, a graduate student in the Hart lab developed an enrichment method that was both highly specific for O-GlcNAc-modified peptides and enhanced the sequencing of these peptides by ETD (23). The method developed by Wang *et al.* introduced PC-PEG-biotin-alkyne, a novel photocleavable biotin tag, onto O-GlcNAc sites and allowed for efficient release of the enriched O-GlcNAc-modified peptides from avidin support. In this workflow, proteins were proteolytically digested, and GlcNAcylated sites were labeled with an azido sugar using UDP-GalNAz and GalT. Following this, GalNAz groups were labeled with the biotin handle using a copper-catalyzed 1,3-dipolar cycloaddition reaction of the free azide on GalNAz with the alkyne molecule containing a biotin and photocleavable linker. O-GlcNAc-modified peptides were enriched by avidin affinity chromatography and released upon brief exposure to UV light (365 nm). An additional and purposeful advantage of using this particular tag is that the photocleavage reaction leaves a basic aminomethyltriazole tag at the site of the O-GlcNAc modification. This resulted in the majority of tryptic peptides existing in z = 3 charge state or higher, thereby enhancing their fragmentation along the peptide backbone during ETD. Under ETD conditions, the dominant fragmentation pathway occurs at the glycosidic linkage, producing oxonium ions at *m*/*z* 300.1 and 503.2. These signature ions are diagnostic for tagged GlcNAc residues and could also be used to detect GlcNAcylated peptides in complex mixtures.

Soon after developing this improved site-mapping method, we applied it to study the role of O-GlcNAcylation in cytokinesis (24). Previously we had demonstrated that OGT localized to the mitotic spindle and midbody, and overexpression of OGT promoted aneuploidy and aberrant spindle formation. We believed that the identification of O-GlcNAcylated mitotic proteins would help pinpoint mechanisms that drive aneuploidy and defective cytokinesis. We purified spindles and midbodies from synchronized cells over-expressing OGT or from control cells and enriched O-GlcNAcylated peptides and phosphorylated peptides from the same samples to study the regulation and interplay of these modifications. We then analyzed samples by ETD in Don’s laboratory and the results were outstanding. From less than 15 μg of spindle and midbody preparation, we identified 450 *O*-GlcNAc–modified peptide pairs and 141 O-GlcNAcylation sites on 64 proteins. We also identified more than 350 phosphorylation sites, representing 190 proteins, from the same sample preparation. Most importantly, over 60 O-GlcNAc-modified proteins with site information provided mechanistic information as to how O-GlcNAc controls mitotic spindle organization, spindle phosphorylation, and midbody dynamics.

By today’s standards, the depth of coverage achieved by Wang *et al.* may seem modest, but at the time, obtaining such results required exceptionally large quantities of protein (25). On many of these proteins, we verified the modification and demonstrated that OGT overexpression affected the protein’s function. For example, we identified 3 O-GlcNAc sites on NuMA1 (Nuclear mitotic apparatus 1), a protein important for centrosome organization, and found that OGT gain of function caused NuMA1 mislocalization. Later, other groups mutated the O-GlcNAc sites on NuMA1 and showed a loss of NuMA1 function (26). Importantly, we presented this data to the scientific community at the FASEB Cell Signaling Conference in the summer of 2010. While phosphorylation cascades were the topic du jour of the conference, our presentation was met with surprise and excitement. Not only did we demonstrate that OGT was critical for mitotic fidelity, but we also identified O-GlcNAcylated proteins involved in regulating spindle dynamics. These results achieved two significant outcomes: they ushered in a tsunami of O-GlcNAc site-mapping papers and increased the credibility of O-GlcNAc as a key regulator of biological function and not a fringe, unimportant sugar modification.

## The Legacy of Donald Hunt’s Contributions to O-GlcNAc Biology and Future Reflections

Since 2008, the Hunt lab has applied MS-based methods to study protein O-GlcNAcylation in both mammalian (27, 28, 29, 30, 31) and plant systems (32, 33, 34, 35, 36). The Hart lab and Hunt lab had many fruitful collaborations, publishing seven papers together in this space (23, 24, 27, 29, 37, 38, 39). Beyond work with the Hart lab, Don applied ETD to identify O-GlcNAc sites on the *Plum pox virus* (PPV) capsid protein (CP) and the work showed that O-GlcNAcylation of PPV CP enhances viral infection in *Arabidopsis thaliana* and showed defects in the stability of the PPV CP. Additional work in *Arabidopsis* showed that O-GlcNAcylation occurs on DELLA transcription factors to fine-tune signaling in response to metabolic signals (34). In 2017, Malaker *et al.* identified O-GlcNAcylated peptides presented by MHC class I molecules in primary leukemia samples and showed that T-cells could kill autologous cells displaying only the modified peptide, suggesting the O-GlcNAc modifications can provide selectivity to antigens that can be targets for immunotherapy (30).

In the past 14 years, thousands of O-GlcNAc sites on greater than 7000 proteins have been mapped. These proteins cover the entire gamut of cellular functions from metabolism, transcription, RNA processing, protein synthesis, signaling, and more. Don’s initial breakthrough with development of ETD was pivotal in bringing O-GlcNAc to the *masses*. Don’s work not only established a link between O-GlcNAc sites and OGT and OGA, but also significantly enhanced our understanding of fundamental cellular mechanisms. Data generated to date support an overarching theory of O-GlcNAc as a cellular rheostat with one central control point (OGT/OGA control of O-GlcNAc cycling) allowing for dynamic adjustment to almost all cellular function in response to environmental changes, though the essentiality of O-GlcNAc continues to challenge the field of study.

Labs from across the globe continue to assign functions to O-GlcNAcylation; however, discerning and interpreting the mechanistic influence of O-GlcNAc remains difficult. With only two proteins processing the addition and removal of the sugar, manipulating either OGT or OGA leads to pleiotropic effects within the cell. Even unimodal approaches like O-GlcNAc site alanine mutants fail to provide enough context to understand an O-GlcNAcylated protein’s function. For example, four sites on the transcription factor FOXO1 were mapped and mutated, but O-GlcNAc levels on FOXO1 did not change (40). OGT is a promiscuous enzyme and is simply labeled S/T next to the mutated sites (40). Moreover, the lack of an O-GlcNAc amino acid mimic limits mutational studies to only a negative regulator. Other PTMs like phosphorylation do not have this problem since Aspartic and Glutamic Acid substitutions can mimic phosphorylation. For O-GlcNAc, the best we can do is to use expressed protein ligation strategies to generate stable O-GlcNAc-modified proteins for functional studies, but this technique only works on a small subset of O-GlcNAc-modified proteins (41). Like 15 years ago, we are again at a pivotal point in O-GlcNAc research. How do we explore the mechanistic role of a modification that does everything, at any time, within the nucleo-cytoplasmic portion of the cell? Once again, we should turn, in part, to the newest advances in mass spectrometry coupled with state-of-the-art O-GlcNAc enrichment methods.

Recent technological and bioinformatic advances are powering a renaissance in mass spectrometry by allowing ultra-fast data-independent acquisition (DIA) to generate deep proteomes and PTMomes in a high-throughput manner for large numbers of samples. However, as discussed above, sensitive and robust O-GlcNAc peptide enrichment methods are needed to improve native O-GlcNAc site mapping in cell lines, tissues, and primary cells. Anti-O-GlcNAc peptide antibodies show promise for simplifying enrichment and identification of O-GlcNAcylation sites, though in practice this method has shown to lack sensitivity when applied to other sample types (42). O-GlcNAcylation research will surely benefit from the continued development of enrichment, and we believe the future is bright. Jerry Hart started the first O-GlcNAc revolution in 1984 when he discovered sugar modification (1). Don Hunt sparked the second revolution in 2004 with the development of ETD (14). We hypothesize that the third revolution in O-GlcNAc research will commence once O-GlcNAc enrichment becomes robust and cost-effective and can be coupled with high-throughput, sensitive mass spectrometry.

## Data Availability

There is no primary data provided within the manuscript.

## Conflict of interest

The authors declare that they have no conflicts of interest with the contents of this article.
